# Solving the Dilemma: Van Wyk-Grumbach Syndrome

**DOI:** 10.7759/cureus.61382

**Published:** 2024-05-30

**Authors:** Sachin Rathod, Shubhada Jajoo, Amardeep Shanoo, Anubha Dande, Divyanshi Kaplish

**Affiliations:** 1 Department of Obstetrics and Gynecology, Datta Meghe Institute of Higher Education and Research, Wardha, IND; 2 Department of Pediatrics, Datta Meghe Institute of Higher Education and Research, Wardha, IND

**Keywords:** thyroxine replacement therapy, van wyk grumbach syndrome, ovarian mass, precocious puberty, hypothyroidism

## Abstract

The Van Wyk-Grumbach syndrome (VWGS) (hypothyroidism, ovarian mass, and precocious puberty) has been extensively documented in the literature as long-term hypothyroidism manifesting as an ovarian mass. The authors of this study describe this entity in a young girl, aged 10, who presented with abdominal pain with a multiloculated ovarian cyst. She was evaluated, and it was discovered that she had delayed bone age, precocious puberty, and a small height. Following her diagnosis of autoimmune thyroiditis and the initiation of thyroxine replacement therapy, the ovarian cysts spontaneously regressed. To avoid needless assessment and surgical mishaps, this entity should be considered in situations of ovarian mass, particularly those with precocious puberty and thyroid disorders.

## Introduction

The most prevalent thyroid condition affecting children is autoimmune hypothyroidism, with Hashimoto thyroiditis accounting for the primary cause. Its incidence in youngsters, particularly in females, is believed to be between 1% and 2% [1]. Children who have hypothyroidism generally show signs of stunted growth and developmental delays; however, infrequently, they may exhibit early signs of puberty. Affected girls can experience an early onset of menstruation, which may occur with or without breast development. Van Wyk and Grumbach initially described this entity, which is often referred to as Van Wyk-Grumbach syndrome (VWGS), in 1960 [2]. The signs and symptoms often manifest gradually and include growth and delayed development, weakness, obesity, dry skin, and bradypsychia. Precocious puberty is a rare symptom of hypothyroidism, as shown in VWGS [3]. It is more common in patients with autoimmune diseases and chromosomal abnormalities, including Down and Turner syndrome [4,5]. We report the premature puberty of a girl who had long-standing, untreated hypothyroidism. Lack of knowledge of this entity might result in oophorectomy, a surgical mishap that has been documented in the literature [6].

## Case presentation

A 10-year-old female child with her mother visited the emergency department with chief complaints that the child had experienced vaginal bleeding for the past two days with pain in the lower abdomen for the last 15 days, which was dull aching, gradual in onset, and progressive in nature, and the bleeding was little, dark colored, and odorless. The pain was not associated with any aggravating or relieving factors. No history of foreign body insertion, sexual abuse, or trauma of any kind was present. Bowel and bladder habits were normal and regular. Sleep and appetite were regular and adequate. There was no significant history of any medical or surgical illness. Family history was not significant. She was the first child born in the birth order, the product of a nonconsanguineous marriage, and delivered vaginally at full term. She experienced her menarche at the age of eight. She had regular, average-flowing menstrual periods and met all her developmental milestones.

Upon examination, she was conscious, well oriented, weighing 24.6 kg, standing 113 cm (<3rd centile), and having a BMI of 19.3 kg/m^2^. Tanner's staging revealed that she had axillary hair and that her breast and pubic hair had sexual maturation scores of B4 and P3, respectively. She was afebrile on touch; a neck examination revealed no lymphadenopathy or goiter. Her blood pressure measured 106/60 mm of mercury, and her pulse measured 90 beats per minute. She appeared pale with no evidence of any edema. A cardiorespiratory examination revealed no obvious abnormality. The abdomen was soft and nontender. The pelvic examination revealed an adnexal mass of approximately 10 x 10 cm on the left and 10 x 6 cm on the right. External genitalia was normal.

Investigations showed normocytic normochromic anemia. Table 1 summarizes the findings of the investigations.

**Table 1:** Laboratory investigation findings β-hCG: Beta-human chorionic gonadotropin; T3: triiodothyronine; T4: thyroxine; AFP: alpha fetoprotein; CEA: carcinoembryonic antigen; CA 125: cancer antigen 125

Sr. No	Investigations	Observed value	Expected value
1	Hemoglobin	9.7	12-16
2	WBC (per cumm)	8900	4000-11,000
3	Platelets (L/cumm)	2.5	1.5-4
4	International normalized ratio (INR)	1.0	0.8-1.1
5	Prothrombin time	11.8	11.9
6	Activated partial thromboplastin time (APTT)	30	29.5
7	Follicle-stimulating hormone (mIU/mL)	4.4	0.3-10
8	Luteinizing hormone (mIU/mL)	0.15	<11.9
9	Prolactin (ng/mL)	24	3-27
10	CA 125 (U/mL)	27.3	<35
11	AFP (IU/mL)	1.4	<40
12	CEA (ng/mL)	1.66	<2.5
13	β-hCG (mIU/L)	0.4	<2.8
14	C-reactive protein	0.16	<0.3
15	Thyroid-stimulating hormone (mIU/L)	499.3	0.5-5.0
16	T3 (pg/dL)	0.9	2.6-5.1
17	T4 (pg/dL)	0.06	0.91-1.66
18	Thyroid peroxidase antibody (IU/ml)	282	<30
19	Antithyroglobulin antibody (IU/ml)	254.3	<10
20	Albumin (g/dL)	4.0	3.5-5.0
21	Total bilirubin	1.0	0.2-1.3
22	Aspartate aminotransferase	41	<50
23	Alanine aminotransferase	31	17-59
24	Serum urea (mg/dL)	21	6-24
25	Serum creatinine (mg/dL)	1.0	0.7-1.2
26	Serum sodium (mEq/L)	141	131-145
27	Serum potassium (mmol/L)	4.1	3.6-5.2
28	Glycated hemoglobin (HBA1c)	4.1	≤5.6
29	Random blood sugar (mg/dL)	90	70-100

**Figure 1:**
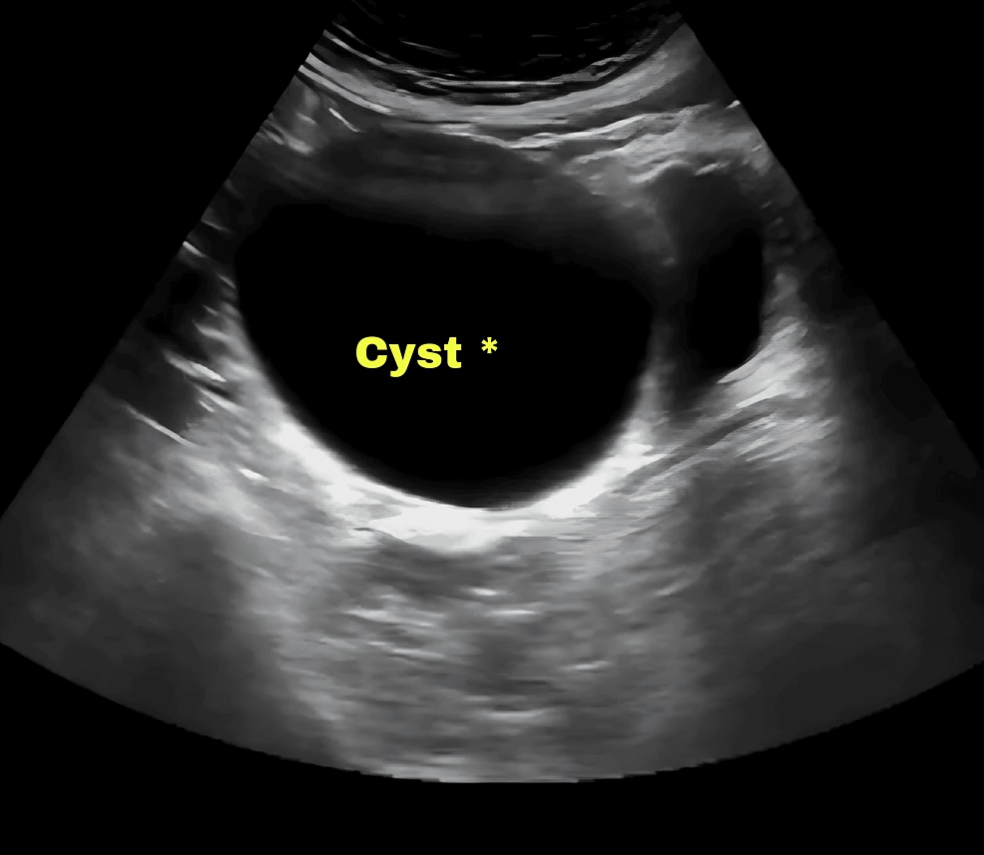
Ultrasound showing the right ovarian cyst of size 10.1 x 6.1 x 5.6 cm

**Figure 2:**
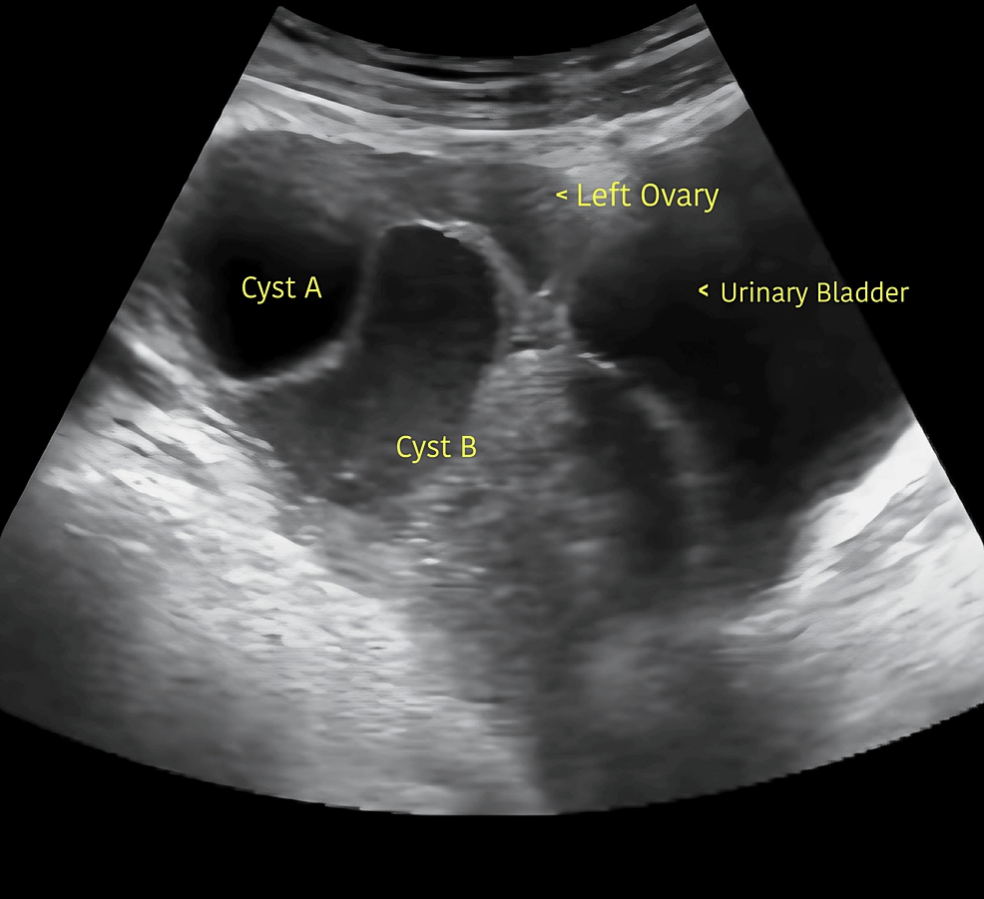
Ultrasound showing the left ovarian cyst A of size 3.2 x 4.3 x 3 cm and cyst B of size 10.2 x 9.3 x 9.6 cm

Urine examination was within normal limits. Ultrasound showed normal kidney size, uterus of 6.0 × 2.8 × 3.8 cm in size, ET of 4 mm, and bilateral enlarged cystic ovaries with the largest cyst in the right ovary of size 10.1 x 6.1 x 5.6 cm (Figure 1) and 10.2 x 9.3 x 9.6 cm in the left ovary (Figure 2) with minimal free fluid in the pouch of Douglas. Her radiological examinations showed that she had eight years and two months of bone age (Greulich and Pyle's atlas).

Ultrasound neck revealed an atrophic thyroid gland. The thyroid profile (thyroid-stimulating hormone (TSH), 499.3 μIU/ml; triiodothyronine (T3), 0.9 pg/ml; thyroxine (T4), 0.06 ng/ml; thyroid peroxidase, 282 IU/ml; antithyroglobulin antibodies, 254.3 IU/ml) demonstrated severe hypothyroidism caused by autoimmune thyroiditis. The presence of hypothyroidism, along with precocious puberty, delayed bone age, and ovarian mass, confirmed VWGS. As per consultation with an endocrinologist, this patient, who was supposed to be taken for emergency laparotomy, was treated with thyroid replacement drugs. T4 (100 + 25 mcg) was started for the child. T4 dosage was adjusted based on TSH levels, which were checked regularly. After a year of therapy, she showed remarkable progress, with her ovarian cyst regressing to 1.2 × 1.3 × 0.7 cm and her irregular bleeding stopped.

## Discussion

In this case, the presence of cystic ovaries and precocious puberty first suggested the potential of an estrogen secreting ovarian tumor. On the other hand, findings of precocious puberty along with delayed bone age narrowed the diagnosis to long-standing hypothyroidism. Ovarian cysts, precocious puberty, delayed bone age, and increased blood TSH values again narrowed it down to the VWGS.

Kendle first documented this condition in 1905, detailing a case involving a nine-year-old girl with fully developed breasts, the onset of menstruation at age five, and exhibiting clinical signs of female cretinism [7]. VWGS is most usually observed in prepubertal females who have myxedematous infiltration of the ovaries, delayed growth, pituitary enlargement, breast development, vaginal bleeding, and ovarian mass [8]. Autoimmune thyroiditis is the most common cause of hypothyroidism in these people [9]. The mechanism behind VWGS has been explained by several theories. Initially, it was described by Van Wyk and Grumbach as a hormonal crossover within the pituitary feedback system. The heterodimeric glycoprotein hormones TSH, FSH, luteinizing hormone (LH), and human chorionic gonadotropin (hCG) have different beta subunits but a common alpha subunit. One glycoprotein may be able to attach to another's receptor due to these molecular similarities. The TSH binding to the ovarian FSH receptor may regulate the excess TSH found in hypothyroidism. Thus, elevated levels of TSH activate the FSH receptor, increasing gonadal size and steroidogenesis [10]. Increased estrogen production in females causes ovarian cysts to occur, as well as breast growth and uterine bleeding without the growth of pubic and axillary hairs.

It is still unclear exactly how precocious puberty develops in these patients. Van Wyk and Grumbach proposed that an excess of multiple hormones is caused by a feedback mechanism that lacks specificity [11]. Considering their level of gonadal stimulation, these patient's blood gonadotropin levels are relatively low. In an in vitro experiment, immunological activity is shown; however, these gonadotropins are not physiologically active [10]. Therefore, the gonadal stimulation observed in severe juvenile hypothyroidism cannot be fully explained by increased gonadotropins alone.

Another theory suggested that in these patients, there is a persistent rise in TSH levels, and the degree of this elevation may have a direct correlation with the tendency to show sexual precocity. Precocity may really be mediated by high levels of TSH in the blood that directly interacts with FSH receptors. The human FSH receptor reaction to TSH has been demonstrated to activate adenylyl cyclase activity by the use of recombinant tools. Using recombinant tools, it has been shown that the human FSH receptor's response to TSH activates adenylyl cyclase activity. Human recombinant TSH, administered at a dosage around 1000 times greater than hFSH, evoked a dose-dependent cyclic AMP response in Chinese hamster ovary (COS-7) cells transfected with the human FSH receptor [10]. This shows that at the very high TSH concentrations found in severe primary hypothyroidism, the very mild FSH-like activity of TSH can be clinically significant.

In addition to this, for patients with severe hypothyroidism, a decrease in thyroid hormone levels may lead to hyperplasia of thyrotropic cells, pituitary hypertrophy, and pituitary stalk compression. These people often have hyperprolactinemia, which is associated with dopamine-mediated disruption of inhibitory tonic control brought on by pituitary stalk compression and elevated thyrotropin-releasing hormone (as a response to declining thyroid hormones). Precocious puberty results from hyperprolactinemia's selective suppression of LH and enhanced FSH production, which lowers the frequency of gonadotropin-releasing hormone pulses [12]. An alternative hypothesis suggests that increased levels of thyrotropin-releasing hormone (TRH) induce thyrotroph hyperplasia, which in turn leads to increase in pituitary size and growth of the sella turcica, often referred as a pituitary adenoma. In those with isosexual precocity, palpable adnexal masses would suggest ovarian malignancies; nevertheless, in all these cases, the bone age is advanced. Therefore, the existence of delayed bone age is one of the most important markers for the diagnosis of VWGS in patients with precocious puberty. The actual etiopathogenesis of the condition needs to be better understood, but the therapeutic strategy is well established. This particular instance is distinct since the youngster was taken to a gynecologist after exhibiting symptoms of an acute abdomen and an ovarian cyst. Lack of knowledge of this entity may result in oophorectomy, a surgical mishap that has been documented in literature, or an unnecessary surgical intervention [13].

Typically, hypothyroidism is linked with delayed puberty, but in VWGS, it manifests as precocious puberty. Additionally, while bone age normally advances in precocious puberty, it is delayed in VWGS. Another mystery is that hypertrichosis is seen in this syndrome, even though hypothyroidism is usually linked to scanty hair growth. Therefore, VWGS can be called the "syndrome of paradoxes."

## Conclusions

This article describes a rare instance of VWGS, which is characterized by precocious puberty, ovarian mass, delayed bone age, and severe hypothyroidism. The hallmark features of this syndrome include irregular bleeding, ovarian cysts, delayed bone age, and breast development. Diagnosis relies on both clinical assessment and additional testing. The main goal of treatment is to replenish depleted thyroid hormone levels through the administration of synthetic thyroid hormone, aiming to attain euthyroidism. While it may be an uncommon occurrence resulting from untreated hypothyroidism, we consider this a significant topic and early suspicion and diagnosis are crucial to prevent invasive additional tests and unnecessary surgeries and to commence suitable treatment for a favorable prognosis.
